# Comparison of clinical characteristics and outcomes between COVID-19 survivors and non-survivors: a retrospective observational study

**DOI:** 10.12688/wellcomeopenres.20919.1

**Published:** 2024-04-17

**Authors:** Diptesh Aryal, Suraj Bhattarai, Sushila Paudel, Subekshya Luitel, Roshni Shakya, Riju Dhakal, Surendra Bhusal, Hem Raj Paneru, Kaveri Thapa, Srijana Kayastha, Karuna Thapa, Sabita Shrestha, Renu Yonjan, Sabin Koirala, Sushil Khanal, Pradip Tiwari, Subhash Prasad Acharya

**Affiliations:** 1 Nepal Intensive Care Research Foundation, Kathmandu, Bagmati Province, Nepal; 2 D'Or Institute for Research and Education, Rio de Janeiro, Brazil; 3 Faculty of Tropical Medicine, Mahidol Oxford Tropical Medicine Research Unit, Mahidol University, Bangkok, Thailand; 4 Global Health Research & Medical Interventions for Development, Kathmandu, Bagmati Province, Nepal; 5 National Academy of Medical Sciences, Bir Hospital, Kathmandu, Bagmati Province, Nepal; 6 Tribhuvan University Teaching Hospital, Kathmandu, Bagmati province, Nepal; 7 Hospital for Advanced Medicine & Surgery, Kathmandu, Bagmati Province, Nepal; 8 Grande International Hospital, Kathmandu, Bagmati Province, Nepal; 9 Civil Service Hospital of Nepal, Kathmandu, Bagmati Province, Nepal

**Keywords:** COVID-19 mortality, clinical characteristics, Nepal

## Abstract

**Background:**

To compare the clinical characteristics of COVID-19 survivors and non-survivors who were transferred from general wards to the critical care units in four tertiary hospitals of Nepal.

**Methods:**

This study utilized electronic data from the National Intensive Care Unit (ICU) registry managed by the Nepal Intensive Care Research Foundation (NICRF). A retrospective observational study was conducted among 78 eligible COVID-19 patients admitted to the intensive care units of four different hospitals between 2020 and 2022. The Mann-Whitney U test was used to compare each continuous variable between the survivors and non survivors, while Pearson's chi-squared test was used to examine the association between each categorical variable and outcome.

**Results:**

Among 78 cases of COVID-19 studied, 29 (37.1%) had died, and 49 (62.8%) had recovered. The median age of the patient was 57.5 years, with a higher percentage of males (66.6%). Hypertension (41%) and diabetes (26.9%) were common comorbidities, whereas pneumonia (41%) and acute respiratory distress syndrome (19.2%) were common complications. Most patients required oxygen therapy (96.2%). Corticosteroids (76.9%) and anticoagulants (83.3%) were commonly administered medications. Median of mean arterial pressure was 85 mm Hg [ IQR- 79.2 - 99.5] in non-survivors and 93.3 mm Hg [IQR- 86.6 - 102.6 ] in survivors which was significantly different between the two groups (p=0.04). Nine of the patients had cardiac dysfunction on ICU admission, of which none survived (p=0.001). Out of ten patients requiring inotropes or vasopressors, only two survived (p=0.001).

**Conclusions:**

Non-survivor COVID patients had lower mean arterial pressure on admission to intensive care units. A higher proportion of patients with cardiac dysfunction and requiring inotropes or vasopressors could not survive.

## Plain language summary

This study aimed to compare the clinical features of COVID-19 patients who survived and those who did not after being transferred from general wards to critical care units in four major hospitals in Nepal. Data from 78 patients, gathered from the National Intensive Care Unit registry, were analysed. Among these patients, 37.1% did not survive, while 62.8% recovered. The analysis revealed that non-survivors had lower mean arterial pressure upon admission to intensive care units compared to survivors. Patients with cardiac dysfunction on admission and those requiring inotropes or vasopressors had a higher likelihood of not surviving. The study emphasizes the importance of understanding these clinical factors in managing severe COVID-19 cases.

## Introduction

The global outbreak of the novel coronavirus disease 2019 (COVID-19), caused by severe acute respiratory syndrome coronavirus 2 (SARS-CoV-2), has presented an unprecedented challenge to healthcare systems worldwide. Since its emergence in late 2019, COVID-19 has rapidly spread, affecting millions of people worldwide, including Nepal. As of September 2023, Nepal has reported 1,003,431 confirmed cases of COVID-19 and 12,031 deaths ^1^. Numerous studies have identified predictors of severity and mortality in COVID-19 patients. Factors such as age, underlying health conditions, and elevated inflammatory markers, including White Blood Cell Counts, Neutrophil-to-Lymphocyte ratio, Serum Glutamic Oxaloacetic Transaminase (SGOT), sodium, and potassium levels, are commonly associated with COVID-19 severity ^2^. Early assessment and monitoring of these laboratory parameters have been shown to be critical in halting disease progression and preventing fatalities ^3^. Recent studies have also focused on molecular biomarkers like IL-6, IL-8, and SP-D, which may offer early indications of disease severity and progression ^4^. Additionally, elevated levels of creatinine, D-dimer, lactate, and potassium in a patient's blood, a low PaO2/FiO2 ratio, and a rapidly increasing alveolar-arterial gradient have been identified as predictors of mortality in critically ill patients ^5^.

COVID-19 can be complicated by severe acute respiratory illness (SARI), often necessitating hospitalization and intensive medical care ^6, 7^. Early recognition and comprehensive management of SARI in COVID-19 patients are crucial for improving outcomes. This study aimed to compare the clinical characteristics of COVID-19 survivors and non-survivors who were transferred from general wards to the critical care units in four tertiary hospitals in Nepal. During COVID-19 surge, these general wards were utilized as isolation units for the infected patients. Patients who were initially admitted in general wards were non-severe COVID-19 patients. These patients were shifted to intensive care units either based on progression to severe COVID or physician discretion. The characteristics of patients presenting with severe COVID-19 in the hospital may differ from the patients who present with non-severe illness and become severe during the hospital stay. So this study only included those patients who were non severe on presentation to the hospitals.

## Methods

### Study design, setting and population

This study employed a retrospective observational design and utilized medical records from four hospitals within the national Intensive Care Unit (ICU) registry managed by the Nepal Intensive Care Research Foundation (NICRF). The registry utilized Case Report Form ^8^ and contained comprehensive information on all patients admitted to the ICU from admission until discharge. The study accessed raw data from the registry covering the period from 2020 to 2022 and considered registered patients who met the following inclusion criteria: (i) Patients aged 18 years or above (ii) having laboratory confirmed SARS-CoV-2 infection; and (iii) initially admitted in general wards. The exclusion criteria were: (i) Patients without lab confirmed COVID-19 (ii) Patients brought to Emergency Department or directly admitted to ICU; and (iii) Patients transferred from another health facility.

**Figure 1.**
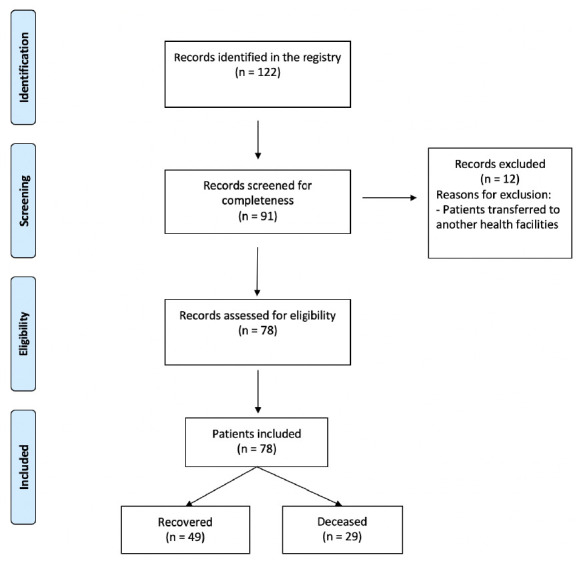
Flowchart of study of the selected patients with COVID-19.

From 122 records initially identified, 91 were screened for completeness ( Figure 1). Out of them, 78 were eligible for inclusion after excluding irrelevant records and patients transferred to another health facility. The final analysis included a total of 49 survivors and 29 non survivors ^8^.

The study collected demographic data, clinical characteristics, laboratory parameters, treatments, and outcomes for all patients.

### Data analysis

The statistical analysis involved tabulating continuous variables as median and interquartile range [IQR]. The Mann-Whitney U test was used to compare continuous variables between the survivors and non-survivors, while Pearson's chi-squared test was used to examine the association between categorical variables and outcome. In cases where the expected frequency in any cell of the contingency table was less than 5, Fisher's exact test was used. The significance level was set at 5%, (alpha=0.05). All statistical analysis was conducted using IBM Statistical Package for the Social Sciences (SPSS) software, version 25.

The study was conducted after obtaining ethical clearance from Nepal Health Research Council (Ref No. 1698/2022) on 23rd January 2023. We received exemption from review of secondary data which was de-identified and did not involve direct patient participation.

## Results

### Socio-demographic and clinical characteristics

Table 1 ^8^ presents the socio-demographics and clinical characteristics of hospitalized patients with COVID-19. The patients had a median age of 57.5 years (IQR 48 - 68.7), with 66.6% being male, and among 29 who died, 20 were males. Upon admission, no significant differences in temperature, heart rate, or respiratory rate were found between the non survivors and survivors. The median peripheral oxygen saturation (Spo2) was lower in both groups (<94%), but this difference was not statistically significant. However, the mean arterial pressure was significantly lower in non-survivor (85 [79.2 - 99.5]) compared to the survivor (93.3 [ 86.6 - 102.6 ]) with a p-value of 0.044.

**Table 1.** Socio-demographics and clinical characteristics of hospitalized patients with COVID-19.

Characteristics	Total (n= 78)	Non- Survivor (n=29)	Survivor (n=49)	P-value
**Age years,** **Median [IQR]**	57.5 [ 48 - 69.25 ]	58 [ 48 - 71 ]	57 [ 48 - 68 ]	0.702
**Sex n (%)**				
Female	26 (33.3)	9 (31)	17 (34.7)	0.740
Male	52 (66.6)	20 (69)	32 (65.3)	
**Vitals at admission**				
Temperature degree Celsius	36.8 [ 36.3 - 37.7 ]	36.7 [ 36.1 - 37.6]	36.8 [ 36.3 - 37.8 ]	0.348
Heart rate beats per minute	95 [ 80 - 110 ]	100 [ 76 - 111 ]	93 [ 80 - 110 ]	0.676
Respiratory rate breaths per minute	24 [ 22 - 28 ]	24.5 [ 22 - 30 ]	24 [ 20 - 25.5 ]	0.059
Peripheral oxygen saturation (SpO2)	91 [ 85.7 - 94 ]	91 [ 83 - 93 ]	90 [ 86 - 94 ]	0.800
Mean Arterial Pressure mm Hg	90 [ 83.3 - 100.3 ]	85 [ 79.2 - 99.5]	93.3 [ 86.6 - 102.6 ]	0.044 *
**Comorbidities n (%)**				
Hypertension	32 (41.0)	11 (37.9)	21 (42.9)	0.669
Diabetes Mellitus	21 (26.9)	9 (31.0)	12 (24.5)	0.529
Renal disease	9 (11.5)	5 (17.2)	4 (8.2)	0.225
COPD/ pulmonary disease	8 (10.3)	2 (6.9)	6 (12.2)	0.452
Hypothyroidism	8 (10.3)	5 (17.2)	3 (6.1)	0.118
Cardiovascular disease	5 (6.4)	2 (6.9)	3 (6.1)	0.893
**Symptoms n (%)**				
Fever	54 (69.2)	19 (65.5)	35 (71.4)	0.585
Shortness of Breath (SOB)	50 (64.1)	18 (62.1)	32 (65.3)	0.773
Cough (non- productive)	33 (42.3)	10 (34.5)	23 (46.9)	0.282
Cough (productive)	19 (24.4)	10 (34.5)	9 (18.4)	0.109
Fatigue/Malaise	9 (11.5)	4 (13.8)	5 (10.2)	0.428
**Vaccination Status n (%)**		.		
Yes	15 (19.2)	5 (17.2)	10 (20.4)	0.190
No	30 (39.7)	8 (27.6)	22 (44.9)	
Unknown	33 (41.0)	16 (55.2)	17 (34.7)	

*p<0.05, n=number of patients, IQR= Interquartile Range, COPD= Chronic Obstructive Pulmonary Disease *(Note: Body Mass Index (BMI) was not calculated due to incomplete entries in the registry)*

In terms of comorbidities, hypertension was the most common comorbidity at 41%, followed by diabetes mellitus (26.9%) and renal disease (11.5%). The most common symptoms were fever (69.2%) and shortness of breath (64.1%). Out of 78 patients, 45 had known vaccination status, and of those 15 (19.2%) were vaccinated.

### Laboratory tests at ICU admission

Table 2 summarizes the laboratory findings of patients upon admission to the ICU. Upon admission to the ICU, the majority of patients (n=48; 61.5%) had decreased lymphocyte counts. Non-survivors had slightly higher levels of AST/SGOT, CRP, LDH, and D-dimer but the differences were not statistically significant. Arterial blood gas analysis also did not reveal any significant differences between the two groups.

**Table 2.** Laboratory tests at ICU admission of hospitalized patients with COVID-19.

Laboratory tests	Total (n= 78)	Non-Survivor (n=29)	Survivor (n=49)	P-value
Haemoglobin (g/dL) (median [IQR])	13.1 [ 10.9 - 14.3 ]	13.8 [ 11.4 - 14.6 ]	12.8 [ 10.5 - 13.8]	0.146
WBC count (x10^9/L)				
< 4.0	5	3	2	0.596
4.0—12.0	42	16	26	
> 12.0	25	9	16	
Lymphocyte count (%)				
<20	48	16	32	0.045 *
20-50	11	7	4	
>50	0	0	0	
Neutrophil count (%)				
<50	1	0	1	0.723
50-79	15	6	9	
>80	38	15	23	
Platelets (10^9/L)				
<150	12	7	5	0.287
150-450	51	18	33	
>450	7	2	5	
ALT/SGPT (U/L)	45 [ 22 - 56.5 ]	48 [ 27 - 54.5 ]	41.5 [ 21.2 - 64.2 ]	0.662
Total bilirubin (mg/dl)	0.7 [ 0.5 - 0.8 ]	0.7 [ 0.54 - 0.8 ]	0.5 [ 0.3- 0.9 ]	0.049
AST/SGOT (U/L)	41 [ 26.7 - 64 ]	47 [ 32.7 - 74.2 ]	35.5 [ 22.2 - 48.2 ]	0.051
Glucose (mg/dl)	130 [ 101 - 170 ]	139 [ 75.5 - 181.2 ]	122 [ 102 - 155 ]	0.355
Urea (mg/dl)				
< 20 mg/dl	23	11	12	0.350
>20	47	17	30	
Na (mequ/l)				
<135	26	12	14	0.666
135-145	45	16	29	
>145	1	0	1	
K (mequ/l)				
<3.5	12	4	8	0.407
3.5—4.5	46	16	30	
>4.5	15	8	7	
Procalcitonin (ng/mL)	0.5 [ 0.1- 4.6 ]	0.9 [ 0.5 - 8.4 ]	0.1 [ 0.1 - 4.6 ]	0.123
CRP (mg/L)	49 [ 15 - 82.5 ]	75 [ 17.5 - 7362 ]	40.5 [ 13.5 - 57.2 ]	0.342
LDH (U/L)	519 [ 270 - 951 ]	855.5 [ 275 - 1000 ]	396 [ 266 - 746 ]	0.301
D-dimer (mg/L)	240.5 [ 23.6 - 557 ]	19.4 [ 2.7 - 980.9 ]	290.7 [ 176 - 538.7 ]	0.307
Ferritin (ng/mL)	1000 [ 428.1 - 1000 ]	1000 [ 724.3 - 1470.5 ]	617 [ 349.9 - 1000 ]	0.164
**Arterial blood gas analysis**				
* pH	7.46 [ 7.41 - 7.48 ]	7.44 [ 7.4 - 7.4]	7.46 [ 7.41 - 7.4 ]	0.458
*pa02/Fi02, mm Hg	128.3 [ 91.6 - 178 ]	123.5 [ 68.8 - 187.4 ]	129 [ 95.1 - 178 ]	0.266
*pC02, mmHg	31.3 [ 28.7 - 34.5 ]	30.4 [ 27.5- 37.2]	31.4 [ 29 - 34.6 ]	0.477
*HC03-, mmol/L	22.5 [ 19.7 - 24 ]	21.9 [ 19.5 - 26.4 ]	22.8 [ 19.8 - 23.9 ]	0.616
**Imaging**				
Chest X-ray infiltrates n (%)	65 (83.3)	24 (82.8)	41 (83.7)	0.999
CT-scan infiltrates n (%)	27 (34.6)	8 (27.6)	19 (38.8)	0.999

*p<0.05, n=number of patients, IQR= Interquartile Range, WBC=White Blood Cell, ALT/SGPT= Alanine Aminotransferase/ Serum Glutamate Pyruvate Transaminase, AST/SGOT= Aspartate Aminotransferase/ Serum Glutamic Oxaloacetic Transaminase, Na=Sodium, K= Potassium, CRP= C-Reactive Protein, LDH= Lactate Dehydrogenase, paO2/FiO2: Ratio of Partial Pressure of Oxygen to Fraction of Inspired Oxygen, pCO2: Partial Pressure of Carbon Dioxide, HCO3-= Bicarbonate , CT= Computed Tomography [ *Note: The laboratory values which had the low number of observations such as interleukins, creatinine, creatine kinase, lactate, activated partial thromboplastin time (APTT), activated partial thromboplastin ratio (APTR), prothrombin time (PT), and international normalized ratio (INR) were not included in the analysis*]

### Complications, treatments, and medications

The most common complications were pneumonia (41%), followed by acute respiratory distress syndrome (ARDS) (19.2%) and cardiac dysfunction (11.5%) ( Table 3). Sepsis and renal failure were less common, occurring in 6.4% and 3.8% of patients, respectively. Notably, all patients with cardiac dysfunction (n=9) died and the analysis showed a significant association between cardiac dysfunction and mortality (p<0.001). Similarly, renal failure also showed significance (p=0.04) but the number of patients with renal failure was low. In terms of treatments, the majority of patients (96.2%) required oxygen therapy. 11.5% required invasive methods, while 29.5% were managed with non-invasive approaches. Regarding medications, corticosteroids and anticoagulants were widely utilized, with usage rates of 76.9% and 83.3% respectively. Antibiotics were given to 80.7% of patients, and 57.7% received antiviral agents, with Remdesivir being the most commonly used (56.4%). Furthermore, out of 10 patients who received inotrope or vasopressors, eight died, which was statistically significant (p=0.001). Lastly, the length of ICU stay was longer for non-survivor (median of 8 [3 - 13.5 ] days) compared to survivor (median of 4 [3 - 9.5] days).

**Table 3.** Complications, treatments, and medications in hospitalized patients with COVID-19.

Characteristics	Total (n= 78) n %	Non-Survivor (n=29)	Survivor (n=49)	P-value
**Complications n(%)**				
Pneumonitis	32 (41.0)	16 (55.2)	16 (32.7)	0.051
ARDS	15 (19.2)	8 (27.6)	7 (14.3)	0.150
Cardiac dysfunction	9 (11.5)	9 (31)	0 (0.0)	<0.001 *
Sepsis	5 (6.4)	4 (13.8)	1 (2.0)	0.061
Renal failure	3 (3.8)	3 (10.3)	0 (0.0)	0.048 *
**Treatment n(%)**				
Oxygen therapy	75 (96.2)	29 (100)	46 (93.9)	0.290
Invasive	9 (11.5)	6 (20.7)	3 (6.1)	0.071
Non-invasive	23 (29.5)	12 (41.4)	11 (22.4)	0.142
Prone positioning	29 (37.2)	9 (31)	20 (40.8)	0.578
**Antiviral medication**	45 (57.7)	18 (62.1)	27 (55.1)	0.547
Remdesivir	44 (56.4)	18 (62.1)	26 (53.1)	0.438
Tocilizumab	3 (3.8)	0 (0.0)	3 (6.1)	-
**Antibiotic medication**	63 (80.7)	26 (89.7)	37 (75.5)	0.137
**Corticosteroids**	60 (76.9)	24 (82.8)	36 (73.5)	0.188
Dexamethasone	54 (69.2)	23 (79.3)	31 (63.3)	0.138
Hydrocortisone	5 (6.4)	1 (3.4)	4 (8.2)	0.646
Methylprednisolone	12 (15.4)	6 (20.7)	6 (12.2)	0.346
**Anticoagulant**	65 (83.3)	24 (82.8)	41 (83.7)	0.999
Enoxaparin	61 (78.2)	22 (75.9)	39 (79.6)	0.700
UFH	3 (3.8)	1 (3.4)	2 (4.1)	-
Rivaroxaban	1 (1.3)	1 (3.4)	0 (0.0)	-
**Inotropes/Vasopressors**	10 (12.8)	8 (27.6)	2 (4.1)	0.001 *
**Length of ICU stay,** **median [IQR], days**	5.5 [ 3 - 10 ]	8 [ 3 - 13.5 ]	4 [ 3 - 9.5 ]	0.121

*p<0.05, n=number of patients, ICU= Intensive Care Unit, IQR= Interquartile Range

## Discussion

In this retrospective observational study, we aimed to explore the clinical characteristics and outcomes of COVID-19 patients who were transferred from the general wards and got admitted to the critical care unit for further management.

The median age of the patients was 57.5 years. While the mean age of COVID-19 patients varies across different studies and locations ^9– 13^, previous reports consistently highlighted the susceptibility of older age groups to severe COVID-19 outcomes ^14– 16^. Moreover, we observed that among those who died, 69% were males, aligning with existing evidence suggesting a higher mortality rate in males compared to females ^17– 21^. Upon admission, we observed no significant disparities in temperature, heart rate, respiratory rate, or symptoms such as fever, shortness of breath, and cough between the survivors and non-survivors, suggesting that initial vital signs and symptoms may not be sufficient markers of disease progression in COVID-19. However, mean arterial pressure (MAP) was different between the two groups, with the non-survivor exhibiting lower MAP values than survivor. This finding may be associated with age and the presence of hypertension, as previous studies have indicated that pre-existing comorbidities can exacerbate COVID-19 severity and increase the risk of mortality ^22– 24^. Notably, hypertension and diabetes mellitus were the most prevalent comorbidities among the hospitalized patients. Other studies have also suggested that MAP is a valuable marker for predicting poor prognosis in COVID-19 patients with hypertension ^25, 26^.

One third of our study patients (5 out of 15) died despite being vaccinated against COVID-19. Although vaccination is considered the best approach to protect oneself from COVID-19 and its complications, it alone may not be effective in preventing severe outcomes including mortality. Factors such as pre-existing comorbidities, breakthrough infections, new variants of the virus, and non-clinical factors like quality of clinical care can contribute to severe outcomes in vaccinated individuals. We also examined the use of biomarkers such as CRP, LDH, and D-dimer, which are frequently used to assess inflammation and coagulopathy in COVID-19 patients ^27^. However, the number of cases in our study were very few to make any definitive conclusions. Use of oxygen, corticosteroids, and anticoagulants was high in our study cases. Oxygen therapy was administered to the majority of patients (96.2%), which was plausible as acute respiratory distress is commonly seen in COVID-19 cases. Antibiotics were used in nearly 70% of cases, regardless of clear indication. Our study has several limitations. Firstly, its retrospective design, relying on data from the National ICU Registry, introduces the potential for data inaccuracies and missing information. Secondly, the relatively small sample size of 78 patients restricts the generalizability of our findings. Thirdly, as we included four ICUs in the study, variations in admission criteria across these ICUs may have influenced patient characteristics and outcomes.

## Conclusion

Our findings provide valuable insights into the clinical characteristics and outcomes of COVID-19 patients seen in health facilities of Nepal. Most of the patients were males, with hypertension being a common comorbidity. Quality of care in general wards can be improved to prevent common complications like pneumonitis, ARDS and cardiac dysfunction which are also the cause of death in COVID-19 patients in low-resource settings. The study also emphasizes proactive monitoring and management of mean arterial pressure (MAP).

## Ethics and consent

The study was conducted after obtaining ethical clearance from Nepal Health Research Council (Ref No. 1698/2022) on 23rd January 2023. We received a waiver of consent from Nepal Health Research Council, Review Board for our study as this is a review of secondary data which was de-identified and did not involve direct patient participation.

## Data Availability

Figshare: Comparison of Clinical Characteristics and Outcomes between COVID-19 Survivors and Non-Survivors: A Retrospective Observational Study. https://doi.org/10.6084/m9.figshare.25040621 ^8^. The project contains the following underlying data: Characteristics and Outcomes_COVID-19_DATA Figshare: Comparison of Clinical Characteristics and Outcomes between COVID-19 Survivors and Non-Survivors: A Retrospective Observational Study. https://doi.org/10.6084/m9.figshare.25040621 ^8^. The project contains the following extended data: CRF-COVID-19-CORE-CRF_2024 and Figure 1_Flow chart of study of the selected patients with COVID-19 Data are available under the terms of the Creative Commons Attribution 4.0 International license (CC-BY 4.0).
