# Supplementary material for: Comparison of clinical characteristics and outcomes between COVID-19 survivors and non-survivors: a retrospective observational study
Source: Wellcome Open Res. 2026 Apr 13;9:204. Originally published 2024 Apr 17. [Version 3] doi: 10.12688/wellcomeopenres.20919.3 (PMC13224356; doi:10.12688/wellcomeopenres.20919.3)
Supplement: Peer review history

# Reviewer report: Reviewer response for version 3

**DOI:** 10.21956/wellcomeopenres.29070.r153112

**Published:** 2026-04-28

**Authors:** Daniel C Gibbons

**Affiliations:** 1 Real-World Data, Measurement & Analytics, Development Sciences, GlaxoSmithKline Research & Development, Stevenage, Hertfordshire, UK

Thanks to the authors for their revisions. No further comments.

Is the work clearly and accurately presented and does it cite the current literature?

Yes

If applicable, is the statistical analysis and its interpretation appropriate?

Partly

Are all the source data underlying the results available to ensure full reproducibility?

No

Is the study design appropriate and is the work technically sound?

Partly

Are the conclusions drawn adequately supported by the results?

Partly

Are sufficient details of methods and analysis provided to allow replication by others?

Yes

Reviewer Expertise:

Epidemiology, biostatistics, COVID-19, clinical medicine.

I confirm that I have read this submission and believe that I have an appropriate level of expertise to confirm that it is of an acceptable scientific standard.

# Reviewer report: Reviewer response for version 2

**DOI:** 10.21956/wellcomeopenres.25779.r113813

**Published:** 2024-12-19

**Authors:** Daniel C Gibbons

**Affiliations:** 1 Real-World Data, Measurement & Analytics, Development Sciences, GlaxoSmithKline Research & Development, Stevenage, Hertfordshire, UK

**Is the study design appropriate and is the work technically sound?**

 Whilst the study design - essentially an observational retrospective registry review - is appropriate, it would be good to understand what the authors mean by 'comprehensive' data. For example, as all cases are lab confirmed, are sequencing data on SARS-CoV-2 available?

 Even if sequencing data are not available, I would recommend that the authors provide summary information on the timing of hospital admission for this cohort of patients (even if it is as broad as, for example, Q2 2021). This may be a reasonable proxy, albeit one prone to ecological bias, to postulate the likely dominant SARS-CoV-2 variant circulating at that point in time.

 Under the assumption that such data are available, I would also recommend that the authors report the delay from symptom onset to hospital and ICU admission for each group.

**If applicable, is the statistical analysis and its interpretation appropriate?**

 Due to the small sample size in this study, I appreciate that multivariate analyses are unlikely to be adequately powered. That being said, I would recommend that the authors note that their point estimates and associated significance are unadjusted.

 As the sample size is small, there is also a risk of type II error. I recommend that the authors perform power calculations to estimate this risk.

 I welcome the addition of the violin plot in Figure 2 to visualise SBP and DBP between groups; I would recommend that some explanatory text is added to aid readers in interpreting box plot component of this graph.

**Are all the source data underlying the results available to ensure full reproducibility?**

 Whilst I applaud the journal's desire to have all source data for a publication available for the purposes of transparency and reproducibility, providing the full raw data for this study would potentially put patient confidentiality at risk.

**Are the conclusions drawn adequately supported by the results?**

 The authors comment that eight of the ten patients who received inotropes and/or vasopressors died - and that this difference was statistically significant. I would recommend that the authors note that such treatment decisions were made in response to the deteriorating clinical state of these patients - and may be a proxy of the presence of shock rather than being associated with poor clinical outcomes in and of themselves.

 The authors also note that one-third of patients who died were vaccinated; however, a greater proportion of survivors were vaccinated compared to non-survivors (20.4% vs. 17.2%) and a substantial proportion of patients had unknown vaccination status (41% overall, 55.2% of non-survivors, 34.7% of survivors). No data presented on whether patients had a complete course of vaccination, or what the timing of their last course of vaccination was relative to symptom onset. In addition to the lack of data accurately describing vaccination status and timing, the small sample size may also risk Type II error.

 Taking these factors into account, I am uncomfortable that the authors suggest *'Although vaccination is considered the best approach to protect oneself... it alone may not be effective in preventing severe outcomes...'* on the basis of their analyses.

 It would be more appropriate to note the lack of data on vaccination administration, its timing and completeness  in this study and that a small sample size, and the associated lack of power, precludes the ability to draw any conclusions on the association of vaccination status and outcomes in this study.

Is the work clearly and accurately presented and does it cite the current literature?

Yes

If applicable, is the statistical analysis and its interpretation appropriate?

Partly

Are all the source data underlying the results available to ensure full reproducibility?

No

Is the study design appropriate and is the work technically sound?

Partly

Are the conclusions drawn adequately supported by the results?

Partly

Are sufficient details of methods and analysis provided to allow replication by others?

Yes

Reviewer Expertise:

Epidemiology, biostatistics, COVID-19, clinical medicine.

I confirm that I have read this submission and believe that I have an appropriate level of expertise to state that I do not consider it to be of an acceptable scientific standard, for reasons outlined above.

# Response

**Published:** 2026-03-28

**Authors:** Subekshya Luitel

**Affiliations:** Nepal Intensive Care Research Foundation, Kathmandu, Central Development Region, Nepal

1. Is the study design appropriate and is the work technically sound? Whilst the study design - essentially an observational retrospective registry review - is appropriate, it would be good to understand what the authors mean by 'comprehensive' data. For example, as all cases are lab confirmed, are sequencing data on SARS-CoV-2 available? Even if sequencing data are not available, I would recommend that the authors provide summary information on the timing of hospital admission for this cohort of patients (even if it is as broad as, for example, Q2 2021). This may be a reasonable proxy, albeit one prone to ecological bias, to postulate the likely dominant SARS-CoV-2 variant circulating at that point in time. Under the assumption that such data are available, I would also recommend that the authors report the delay from symptom onset to hospital and ICU admission for each group.

**Response:  **Thank you for the comment. We have clarified the registry variables in the Methods section by expanding “comprehensive” with a description of included data (demographic, clinical, laboratory, treatment, and outcome variables). SARS-CoV-2 sequencing data were not available. To provide temporal context, we have added admission timing: 31 (39.7%) in 2020, 25 (32.1%) in 2021, and 22 (28.2%) in 2022. We have also added a statement in the Discussion noting that admissions across multiple years likely reflect exposure to different circulating variants. Data on symptom onset to hospital/ICU admission were not available and are acknowledged as a limitation.

 2.  If applicable, is the statistical analysis and its interpretation appropriate? Due to the small sample size in this study, I appreciate that multivariate analyses are unlikely to be adequately powered. That being said, I would recommend that the authors note that their point estimates and associated significance are unadjusted. As the sample size is small, there is also a risk of type II error. I recommend that the authors perform power calculations to estimate this risk. I welcome the addition of the violin plot in Figure 2 to visualise SBP and DBP between groups; I would recommend that some explanatory text is added to aid readers in interpreting the box plot component of this graph.

**Response:** Thank you for these valuable suggestions. We have clarified in the Methods section that all analyses were unadjusted and no multivariable analysis was performed due to the limited sample size. We have also added in the limitations that the small sample size increases the risk of type II error and that findings should be interpreted with caution. Formal power calculations were not performed and this has been acknowledged as a limitation. We have added explanatory text to Figure 2 to aid interpretation of the violin plot and its embedded box plot.

 3. Are all the source data underlying the results available to ensure full reproducibility? Whilst I applaud the journal's desire to have all source data for a publication available for the purposes of transparency and reproducibility, providing the full raw data for this study would potentially put patient confidentiality at risk.

**Response: **The dataset used in this study has been shared in Figshare in accordance with journal requirements. All data were de-identified prior to sharing to ensure patient confidentiality, and no personally identifiable information is included.

 4. Are the conclusions drawn adequately supported by the results? The authors comment that eight of the ten patients who received inotropes and/or vasopressors died - and that this difference was statistically significant. I would recommend that the authors note that such treatment decisions were made in response to the deteriorating clinical state of these patients - and may be a proxy of the presence of shock rather than being associated with poor clinical outcomes in and of themselves. The authors also note that one-third of patients who died were vaccinated; however, a greater proportion of survivors were vaccinated compared to non-survivors (20.4% vs. 17.2%) and a substantial proportion of patients had unknown vaccination status (41% overall, 55.2% of non-survivors, 34.7% of survivors). No data presented on whether patients had a complete course of vaccination, or what the timing of their last course of vaccination was relative to symptom onset. In addition to the lack of data accurately describing vaccination status and timing, the small sample size may also risk Type II error. Taking these factors into account, I am uncomfortable that the authors suggest 'Although vaccination is considered the best approach to protect oneself... it alone may not be effective in preventing severe outcomes...' on the basis of their analyses. It would be more appropriate to note the lack of data on vaccination administration, its timing and completeness  in this study and that a small sample size, and the associated lack of power, precludes the ability to draw any conclusions on the association of vaccination status and outcomes in this study.

**Response: **Thank you for this valuable observation. We have revised the interpretation to clarify that the use of inotropes and vasopressors likely reflects underlying shock and clinical deterioration rather than being independently associated with mortality. We have also revised the interpretation of vaccination findings. Due to incomplete data on vaccination status, timing, and dosage, as well as the small sample size and associated limited power, we have removed causal statements and now state that no conclusions can be drawn regarding the association between vaccination and outcomes in this study. These limitations have been explicitly acknowledged in the revised manuscript.      

# Reviewer report: Reviewer response for version 2

**DOI:** 10.21956/wellcomeopenres.25779.r110433

**Published:** 2024-11-11

**Authors:** Tomokazu Konishi

**Affiliations:** 1 Akita Prefectural University, Akita, Japan

As I am seeing the data, Peripheral Oxygen Saturation- ICU Admission and Respiratory Rate per minute - ICU Admission has clear differences between the groups. Please make violin plots for those classes, not the blood pressures. Those could be convenient tools.

Is the work clearly and accurately presented and does it cite the current literature?

Yes

If applicable, is the statistical analysis and its interpretation appropriate?

Yes

Are all the source data underlying the results available to ensure full reproducibility?

Yes

Is the study design appropriate and is the work technically sound?

Yes

Are the conclusions drawn adequately supported by the results?

Partly

Are sufficient details of methods and analysis provided to allow replication by others?

Yes

Reviewer Expertise:

bioinformatics

I confirm that I have read this submission and believe that I have an appropriate level of expertise to confirm that it is of an acceptable scientific standard.

# Reviewer report: Reviewer response for version 1

**DOI:** 10.21956/wellcomeopenres.23145.r100037

**Published:** 2024-09-23

**Authors:** Tomokazu Konishi

**Affiliations:** 1 Akita Prefectural University, Akita, Japan

There has always been concern that patients with heart disease might be more likely to develop severe COVID, and this is a straightforward result of that. We believe that this is an important result for future care. The data is reliable and the way it was compiled is highly valid. On that basis, I have only one request to make

 Regarding the mean arterial pressure:

 The mean arterial pressure: There was probably a large variation between individual patients. The way Table 2 is presented only shows the average value. This is a great pity. Some patients would have been hypertensive, while others would have had significantly lower blood pressure. These should be considered separately. Hypertension is considered to be one of the symptoms of COVID, but was it mainly patients with heart disease whose blood pressure fell?

 One additional figure should be added for this. I would like you to create a violin plot for each systolic and diastolic blood pressure, based on the data from each patient. If this is difficult, then please create histograms for survivors and deaths for each of them and show them.

 That's all I have to ask for.

 Incidentally, but there are reports that vaccines are no longer lowering mortality.

https://www.mdpi.com/2673-8112/4/2/12

 There are also a number of reports, particularly for mRNA vaccines, that these induce IgG4, which in turn impairs immunity. This result confirms such reports.

Is the work clearly and accurately presented and does it cite the current literature?

Yes

If applicable, is the statistical analysis and its interpretation appropriate?

Yes

Are all the source data underlying the results available to ensure full reproducibility?

Yes

Is the study design appropriate and is the work technically sound?

Yes

Are the conclusions drawn adequately supported by the results?

Partly

Are sufficient details of methods and analysis provided to allow replication by others?

Yes

Reviewer Expertise:

bioinformatics

I confirm that I have read this submission and believe that I have an appropriate level of expertise to confirm that it is of an acceptable scientific standard, however I have significant reservations, as outlined above.

# Response

**Published:** 2024-11-05

**Authors:** Subekshya Luitel

**Affiliations:** Nepal Intensive Care Research Foundation, Kathmandu, Central Development Region, Nepal

1. Regarding the mean arterial pressure:

 The mean arterial pressure: There was probably a large variation between individual patients. The way Table 2 is presented only shows the average value. This is a great pity. Some patients would have been hypertensive, while others would have had significantly lower blood pressure. These should be considered separately. Hypertension is considered to be one of the symptoms of COVID, but was it mainly patients with heart disease whose blood pressure fell?

**Response: **There are only five patients with cardiac disease. Hence, it could not be assessed if only the patients with cardiac disease had a fall in blood pressure.

 2. One additional figure should be added for this. I would like you to create a violin plot for each systolic and diastolic blood pressure, based on the data from each patient. If this is difficult, then please create histograms for survivors and deaths for each of them and show them.

**Response: **We have added Figure 2, which displays the violin plot of systolic and diastolic blood pressure distribution between COVID-19 survivors and non-survivors.
